# The Cytopathogenic BVDV Core Protein Binds with ASC-Enhance the Assembly of Inflammasome Complex and GSDMD-Mediated Pyroptosis

**DOI:** 10.3390/vetsci13070673

**Published:** 2026-07-10

**Authors:** Ning He, Hongming Zhou, Jiaming Yang, Jiying Yin, Qi Wang, Zitong Jing, Yang Liu, Yuxin Kong, Fanli Zeng, Jianming Li, Naichao Diao, Kun Shi, Rui Du

**Affiliations:** 1 College of Animal Medicine, College of Animal Science and Technology, Jilin Agricultural University, Changchun 130118, China; 18043212558@163.com (N.H.); 18734248326@163.com (J.Y.); yinjiying1103@163.com (J.Y.); wangqi3820@163.com (Q.W.); 18654513391@163.com (Z.J.); violet.ly.lucky@163.com (Y.L.); 2 Agriculture College, Yanbian University, Yanji 133002, China; xm757137@icloud.com (H.Z.); 0000006032@ybu.edu.cn (Y.K.); 3 College of Chinese Medicinal Materials, Jilin Agricultural University, Changchun 130118, China; zengfanli@jlau.edu.cn (F.Z.); lijianming4773@163.com (J.L.); diaonaichao@jlau.edu.cn (N.D.)

**Keywords:** bovine viral diarrhea virus, NLRP3 inflammasome, ASC, pyroptosis

## Abstract

Bovine viral diarrhea virus (BVDV) infection is associated with inflammatory responses, but the mechanisms underlying inflammasome activation remain unclear. In this study, Madin–Darby bovine kidney (MDBK) cells were used to compare the inflammatory responses induced by the cytopathogenic NADL strain and the non-cytopathogenic TC strain. Both strains significantly increased IL-1β and IL-18 production and promoted NLRP3 inflammasome assembly, indicating activation of upstream inflammasome signaling. However, only the NADL strain markedly induced Gasdermin D (GSDMD) cleavage, membrane pore formation, lactate dehydrogenase release, and pyroptotic cell death. Further mechanistic analyses demonstrated that the NADL core protein C interacted with the inflammasome adaptor ASC, thereby facilitating NLRP3 inflammasome assembly and enhancing inflammatory cytokine secretion. Silencing ASC significantly impaired inflammasome activation and pyroptosis induced by protein C, confirming its essential role in this process. Collectively, these findings demonstrate that the cytopathogenic BVDV NADL strain activates ASC-dependent NLRP3 inflammasome signaling and GSDMD-mediated pyroptosis through its core protein C, providing a molecular explanation for the distinct inflammatory responses and pathogenic outcomes associated with different BVDV biotypes.

## Simple Summary

Bovine viral diarrhea virus (BVDV) causes substantial economic losses to the global cattle industry. Although inflammation is a hallmark of BVDV infection, its underlying mechanisms remain poorly understood. In this study, we found that both cytopathogenic and non-cytopathogenic BVDV strains activated inflammatory signaling in bovine kidney cells, whereas only the cytopathogenic strain induced pyroptosis, an inflammatory form of programmed cell death. Mechanistically, the viral core protein C interacted with ASC, thereby promoting NLRP3 inflammasome activation and the release of pro-inflammatory cytokines. These findings provide new insights into the inflammatory pathogenesis of BVDV infection and the biological differences between BVDV biotypes.

## 1. Introduction

Bovine viral diarrhea (BVD), as a major infectious disease affecting cattle worldwide, has long imposed substantial economic burdens on the cattle industry [1]. Its pathogen, bovine viral diarrhea virus (BVDV), has complex biological characteristics, including the existence of different biotypes, the capacity to cross the placental barrier, and the capacity to establish persistent infections, which result in sustained viral circulation in natural populations and hinder effective eradication by conventional control measures [2,3]. BVDV infection can cause the host to exhibit a series of clinical symptoms such as fever, diarrhea, reproductive disorders, and lethal mucosal disease, and the pathological process is often accompanied by pronounced inflammatory responses, such as leukopenia and tissue inflammatory infiltration [4]. This suggests that host immune responses, particularly inflammatory responses, play a critical role in BVDV pathogenesis [5,6]. However, the inflammatory responses and infection outcomes caused by different biotypes of BVDV, show significant differences, and their underlying molecular mechanisms remain poorly understood.

BVDV belongs to the family Flaviviridae and the genus Pestivirus [7]. When the virus enters host cells, its single-stranded positive-sense RNA genome is first translated to produce a large polyprotein precursor, which is then proteolytically processed into mature viral proteins by host signal peptidases and viral proteases [8]. These proteins are classified into structural proteins (C, Erns, E1, and E2) and non-structural proteins (Npro, p7, NS2/3, NS4A, NS4B, NS5A, and NS5B) [9,10,11]. These viral proteins not only constitute key components of the viral replication complex, directly participating in genome replication and virion assembly, but also modulate host cellular signaling pathways and immune responses through diverse mechanisms, thereby contributing to the molecular basis of BVDV pathogenesis [12].

Based on whether it can cause significant cytopathogenic effects in cultured cells in vitro, BVDV is generally classified into two classical biotypes: cytopathogenic (CP) and non-cytopathogenic (NCP). This difference in biological characteristics is closely associated with infection outcomes in vivo [13,14]. Epidemiological evidence indicates that NCP BVDV is the predominant strain circulating in natural populations, and after infecting pregnant dams, it can cross the placental barrier to infect the fetus [15]. Owing to the immature state of the fetal immune system, it recognizes the virus as a self component, thereby forming immune tolerance, resulting in calves born as persistently infected (PI), animals that harbor and shed the virus throughout life [16]. PI animals serve as the primary reservoirs for sustained BVDV transmission within herds. In contrast, CP BVDV typically cannot establish persistent infection; however, superinfection of PI animals with CP BVDV often triggers fatal mucosal disease [17]. Although this macro-level pathogenic pattern has been recognized for many years, the molecular mechanisms underlying the differences in biological behavior between CP and NCP BVDV, especially their differential regulation of host inflammatory responses, still remain poorly understood.

The Madin–Darby bovine kidney (MDBK) cell is highly susceptible to different BVDV biotypes, and can stably support efficient viral replication [18,19]; thus, it has become a well-established in vitro model for studying the biology and pathogenic mechanisms of BVDV infection. With the help of this model, previous studies have initially revealed the differences between CP and NCP BVDV in viral replication kinetics and protein accumulation, among other aspects [20]. However, whether these two biotypes determine infection outcomes toward lytic clearance or persistent survival by differentially regulating host inflammatory responses and cell death programs remains to be systematically elucidated at the mechanistic level.

Pyroptosis is an important critical innate immune defense mechanism of the host against viral infection; inflammasomes play a central regulatory role in this process [21]. The NLRP3 inflammasome consists of the sensor molecule NLRP3, the adaptor ASC, and the downstream effector pro-Caspase-1. Its activation can promote the self-cleavage activation of Caspase-1, thereby mediating GSDMD cleavage, forming membrane pores and inducing pyroptosis, while also driving the maturation and secretion of IL-1β and IL-18 [22,23]. Current research indicates that various viruses can activate the NLRP3 pathway by disturbing ion homeostasis, inducing mitochondrial damage, or directly acting on inflammasome components, thereby triggering GSDMD-dependent pyroptosis and further amplifying inflammatory responses and tissue damage [21,24,25]. However, there is still a lack of systematic research on whether BVDV infection of MDBK cells can activate inflammasomes and induce pyroptosis, and on the differential effects of different BVDV biotypes in the above processes. More importantly, whether the BVDV-encoded proteins directly participate in the regulation of inflammasomes and their potential molecular mechanisms has not yet been elucidated.

This study employed MDBK cells as a model to compare the effects of the cytopathogenic BVDV strain NADL and the non-cytopathogenic strain TC on inflammasome activation and pyroptosis, and to analyze the key mechanisms by which BVDV-encoded viral proteins mediate inflammasome activation, aiming to elucidate the pathogenic mechanism of BVDV from the perspective of inflammatory immunity and programmed cell death and thereby provide theoretical support for the development of prevention and control strategies targeting the inflammasome pathway.

## 2. Materials and Methods

### 2.1. Cells Culture and Virus Infection

Madin–Darby bovine kidney (MDBK, Bio-68289) cells were obtained from the Cell Bank of Type Culture Collection of Chinese Academy of Sciences (TCCCAS) and grown in Dulbecco’s Modified Eagle Medium (DMEM, Gibco, Grand Island, NY, USA) supplemented with 10% fetal bovine serum (Gibco, Grand Island, NY, USA), at 37 °C in a humidified incubator containing 5% CO_2_. The BVDV NADL and TC strains used in this study were previously isolated, identified, and preserved in our laboratory. The cytopathogenic (CP) BVDV NADL strain exhibits a cytopathogenic phenotype in MDBK cells, whereas the non-cytopathogenic (NCP) BVDV TC strain exhibits a non-cytopathogenic phenotype. Viral stocks were propagated in MDBK cells and titrated prior to use [26].

### 2.2. Antibodies and Reagents

Goat anti-mouse IgG H&L (FITC) (ab6785, Abcam, Cambridge, UK); anti-F-actin antibody (bs-1571R, Bioss, Beijing, China); Coralite488-conjugated Goat Anti-Rabbit IgG (H + L) (SA00013-2), and Coralite594-conjugated Goat Anti-Mouse IgG (H + L) (SA00013-3) were from Proteintech (Rosemont, IL, USA); Caspase-1 Rabbit pAb (A0964), GAPDH Rabbit pAb (AC027), NLRP3 Rabbit mAb (A24294), ASC Rabbit mAb (A24165), and GSDMD (Full Length + N terminal) Rabbit pAb (A24476) were from ABclonal (Shanghai, China); Poly I:C (42-875-0) was purchased from Gibco (Grand Island, NY, USA) and was used as a general inflammatory stimulus to induce antiviral inflammatory responses, serving as a positive control for inflammation rather than as a specific activator of the canonical NLRP3 inflammasome pathway.

### 2.3. Lentiviral Packaging

Based on the published BVDV NADL strain sequences in GenBank, specific primers were designed, and PCR was used to amplify the full-length coding sequences of 11 genes: Npro, C, Erns, E1, E2, NS2, NS3, NS4A, NS4B, NS5A, and NS5B. All primers were synthesized by Sangon Biotech Co., Ltd. (Shanghai, China). The PCR amplification products were recovered and purified after identification by agarose gel electrophoresis, and then subjected to double digestion using the corresponding restriction endonucleases. Subsequently, the target fragment was ligated into the pLenti-EF1a-EGFP-P2A-Puro-CMV-MCS-3Flag lentiviral expression vector, which had been linearized with the same restriction endonucleases. The ligation product was transformed into DH5α-competent Escherichia coli, and positive clones were screened on LB solid medium containing ampicillin. After extracting the recombinant plasmid, the correctness of the inserted sequence was verified by DNA sequencing. Lentiviral packaging was carried out using a three-plasmid cotransfection system. The recombinant expression plasmid, the packaging plasmid psPAX2, and the envelope plasmid pMD2.G were mixed in a 3:2:1 ratio and diluted using Opti-MEM (Gibco, Grand Island, NY, USA) medium. At the same time, the PEI 40K (Servicebio, Wuhan, China, G1802-1ML) transfection reagent was diluted with Opti-MEM, then thoroughly mixed with the plasmid solution and incubated at room temperature for 15 min to form DNA-PEI complexes. Subsequently, the complexes were added to HEK293T cells for transfection. After 18 h of transfection, this was replaced with fresh complete medium and culturing continued. At 48 h post-transfection, the cell culture supernatant was collected, centrifuged to remove cell debris, and filtered through a 0.45 μm membrane for sterilization. Finally, the obtained lentivirus supernatant was aliquoted and stored at −80 °C for subsequent cell infection and functional validation experiments.

### 2.4. Immunofluorescence

MDBK cells were cultured on coverslips in a 48-well plate at a density of 2 × 10^5^ cells/well, and then infected with BVDV NADL and TC strains at an MOI of 1. After 36 h of incubation, the culture medium was aspirated, and cells were washed thoroughly, fixed with 4% paraformaldehyde (PFA) for 10 min, and then permeabilized with 0.5% Triton X-100 in PBS for 8 min. Subsequently, cells were blocked with 3% bovine serum albumin (BSA, Source Leaf, Shanghai, China), followed by incubation with primary antibodies targeting NLRP3, ASC, or Caspase-1 (1:100), overnight at 4 °C. After washing with PBS, secondary antibodies conjugated with Coralite488 and 594 (1:200) were added and incubated for 2 h in the dark; then, cell nuclei were stained with DAPI (4′,6-diamidino-2-phenylindole) and slides were mounted using FluoroGuard Antifade Mounting Medium (Invitrogen). Finally, images were observed and acquired using a laser confocal microscope (Leica STELLARIS 5, Wetzlar, Germany). Finally, Image-Pro Plus 6.0 software (Media Cybernetics, Rockville, MD, USA) was used to quantify the fluorescence intensity of the immunofluorescence signals. The mean fluorescence intensity was calculated from randomly selected fields and used for statistical analysis.

### 2.5. LDH

MDBK cells were seeded at 2 × 10^5^ cells/mL in 6-well plates and cultured for 24 h, then infected with NADL and TC strains at an MOI of 1, with uninfected negative control and Poly I-positive control groups set up at the same time. After 36 h of infection, the cell culture supernatant was collected and a commercial LDH kit (Panteze Bio, Shenzhen, China) was used for testing according to the manufacturer’s instructions. After incubation, washing, and color development according to the kit instructions, the absorbance was measured at 450 nm using a microplate reader. LDH release levels were used to assess cell membrane damage and the extent of cell death.

### 2.6. RNA Extraction and Quantitative PCR (qPCR)

Total RNA was extracted from BVDV-infected MDBK cells at 36 h post-infection using an RNA extraction kit (TaKaRa, Maebashi, Japan) following the manufacturer’s instructions. Following cell lysis and centrifugation, the supernatant was collected, mixed with 70% ethanol, and then transferred to a purification column for sequential washing and centrifugation, and then DEPC water was added to the column. Following incubation at room temperature for 5 min, the sample was centrifuged at 12,000 rpm for 2 min to elute RNA. The purified RNA was quantified and stored at −80 °C.

Genomic DNA was subsequently removed using the PrimeScript RT Reagent Kit with gDNA Eraser (TaKaRa, Maebashi, Japan), and reverse transcription was performed following the manufacturer’s protocol. The protocol involved gDNA removal at 42 °C for 5 min, before cDNA was synthesized at 37 °C for 15 min, and the enzyme was inactivated by incubating at 85 °C for 5 s. Using cDNA as a template, the real-time quantitative PCR mixture was prepared using TB Green™ Premix Ex Taq™ II (TaKaRa, Maebashi, Japan), The 2^−ΔΔCt^ method was used to calculate the relative expression of mRNA. The primers used for real-time PCR are mentioned.

### 2.7. TEM

MDBK cells were seeded onto coverslips in 6-well plates at a density of 1 × 10^5^ cells per well, and infected with BVDV NADL strain and TC strain at an MOI of 1, respectively. After 36 h, cells were gently scraped using a pre-chilled cell scraper, centrifuged at 1000 rpm, and fixed overnight at 4 °C using 2.5% glutaraldehyde (Yuan Ye, Zhongshan, China). Afterwards, following three washes with phosphate-buffered saline (PBS, pH 7.4), the samples were post-fixed in 1% osmium tetroxide for 1 h and dehydrated through a graded series of ethanol and acetone. Subsequently, they were gradually infiltrated with a mixture of acetone and epoxy resin, embedded, and polymerized to fully cure the resin. Ultrathin sections were subjected to double-staining with uranyl acetate and lead citrate, followed by an examination of cellular ultrastructure using transmission electron microscopy (TEM. FEI Tecnai F20 FEI, Chicago, IL, USA).

### 2.8. ELISA

Bovine IL-1β and IL-18 concentrations in culture supernatants were quantified using ELISA. MDBK cells were seeded into 6-well plates and cultured until they reached approximately 80% confluence, then infected with BVDV NADL and TC strains at an MOI of 1 to establish infection models. After 12, 24, 36, 48 h of infection, the cell supernatant was collected. Standard solutions were prepared and standard curves were drawn according to the kit instructions (Panteze Bio, Shenzhen, China). During detection, the sample was added to the well after being diluted in proportion, and the plate was incubated with the standard product at 37 °C for 1 h. Following plate washing, the enzyme-labeled secondary antibody was added and incubated again for 1 h, followed by repeating the washing steps. Subsequently, the chromogenic substrate solution was added, and the reaction was terminated after 15 min in the dark. Finally, the samples were measured using the Hidex Sense microplate reader (Synergy HTX, Santa Clara, CA, USA) at 450 nm, and the concentrations of IL-1β and IL-18 in the samples were calculated according to the standard curve.

### 2.9. Immunoblotting

After 36 h of treatment, the culture medium was aspirated, and MDBK cells were harvested for total protein extraction. Protein concentration was quantified using a bicinchoninic acid (BCA) assay kit (Beyotime, Shanghai, China); Equal amounts of protein were mixed with 4 × loading buffer and denatured by heating at 100 °C for 10 min. Protein samples were separated on sodium dodecylsulfate–polyacrylamide gel electrophoresis (SDS-PAGE) (Seven, China) and transferred to a polyvinyl difluoride (PVDF) membranes, and membranes were blocked with 5% skim milk in TBST (0.05% Tween-20) for 2 h at room temperature, and incubated overnight at 4 °C with antibodies diluted as indicated: GAPDH (1:2000), Caspase-1 (1:3000), NLRP3 (1:1500), and GSDMD (1:3500). Following three washes with TBST, the membranes were incubated with HRP-conjugated secondary antibodies (goat anti-mouse IgG (H+L) (1:10,000) or goat anti-rabbit IgG (H+L) (1:20,000)) at 37 °C for 2 h and visualized using an ultra-sensitive ECL kit, and protein band signals were captured using an imaging system. Finally, Image-Pro plus 6.0 software (Media Cybernetics, Rockville, MD, USA) was used to analyze the signal intensity of the protein bands.

### 2.10. Co-Immunoprecipitation Assay (Co-IP)

Upon reaching 80% confluency, MDBK cells were transfected with a plasmid expressing Flag-tagged BVDV C protein, with an empty vector as a negative control. After 36 h of transfection, the cells were washed with pre-cooled PBS and then lysed on ice with IP lysis buffer containing protease inhibitors (Beyotime, Shanghai, China) on ice. The lysates were centrifuged at 12,000× *g* at 4 °C for 15 min to collect the supernatant, and the protein concentration was measured. An equal amount of protein was incubated with anti-Flag M2 affinity gel or magnetic beads (Beyotime, Shanghai, China) at 4 °C overnight. Following incubation, the beads/gel were washed, and 5 × SDS loading buffer was added to boil and elute the immunoprecipitated proteins. Immunoprecipitated products and input samples were separated by SDS-PAGE and transferred to a PVDF membrane. After blocking, primary antibodies against NLRP3, ASC, Caspase-1, and Flag were incubated at 4 °C overnight. The membrane was incubated with HRP-conjugated secondary antibodies, and visualized with ECL chemiluminescence reagents. The results were analyzed by image software.

### 2.11. Small Interfering RNA (siRNA)

Small interfering RNA targeting ASC (si-ASC) and non-targeting negative control siRNA (si-NC) were synthesized by GenePharma (Suzhou, China). The si-ASC sequences were: sense, 5′-GCACUAGUUCGGACAAGAUTT-3′; antisense, 5′-AUCUUGUCCCAACUAGUGCTT-3′. Cells in the logarithmic growth phase were digested, counted, seeded into 6-well plates, and cultured overnight at 37 °C with 5% CO_2_. When cell confluence reached approximately 60%, transfection was performed. For each well, 15 pmol siRNA was mixed with 8.5 μL buffer, followed by addition of 1.5 μL Plus transfection reagent to form siRNA/Plus complexes. The complexes were added dropwise to cells and evenly distributed by gentle rocking. After 72 h, ASC knockdown efficiency was assessed by qRT-PCR.

### 2.12. Statistical Analysis

All data are expressed as the mean ± standard deviation (mean ± S.D.), which was determined using a one-way analysis of variance (ANOVA) and Bonferroni’s post hoc test. The statistical data are shown as graphs generated using GraphPad Prism 8.0.2 software (GraphPad Software Inc., San Diego, CA, USA): * denotes *p* < 0.05; ** denotes *p* < 0.01; *** denotes *p* < 0.001.

## 3. Results

### 3.1. BVDV Infection Induces NLRP3 Inflammasome-Mediated Inflammatory Responses

**Figure 1:**
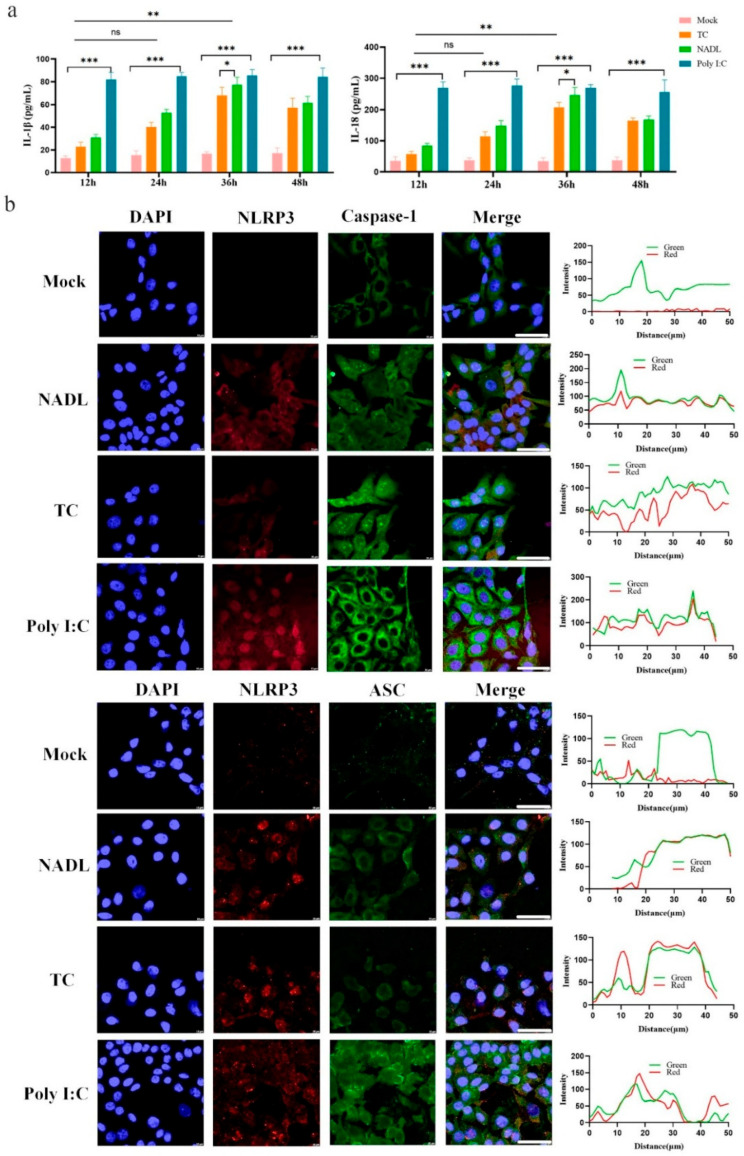
BVDV infection induce NLRP3 inflammasome-mediated inflammatory responses. (**a**) MDBK cells were infected with the cytopathogenic BVDV NADL strain or the non-cytopathogenic BVDV TC strain at an MOI of 1. Pro-inflammatory cytokine secretion levels were measured at 12 h, 24 h, 36 h, and 48 h post-infection. (**b**) Co-localization of ASC and Caspase-1 with NLRP3 after 36 h of IFA detection. Scale bar: 50 μm. Data are expressed as mean ± SD. * *p* < 0.05; ** *p* < 0.01; *** *p* < 0.001.

To investigate whether BVDV infection of MDBK cells induces an inflammatory response, ELISA assays were performed to measure IL-1β and IL-18 secretion levels following infection with the cytopathogenic NADL strain and the non-cytopathogenic TC strain. Results demonstrated that both BVDV NADL and TC strains significantly induced pro-inflammatory cytokine secretion (Figure 1a). Given that the NLRP3 inflammasome comprises NLRP3, ASC, and Caspase-1, we further employed laser confocal microscopy to detect the expression and activation status of its key components, thereby assessing whether the NLRP3 inflammasome was activated. Results showed that both the BVDV NADL and TC strains exhibited colocalization of ASC and Caspase-1 with NLRP3 in the cytoplasm (Figure 1b). Collectively, these results indicate that BVDV infection activates the NLRP3 inflammasome and contributes to the inflammatory response.

### 3.2. BVDV NADL Induces NLRP3 Inflammasome Activation Not TC Strain

**Figure 2:**
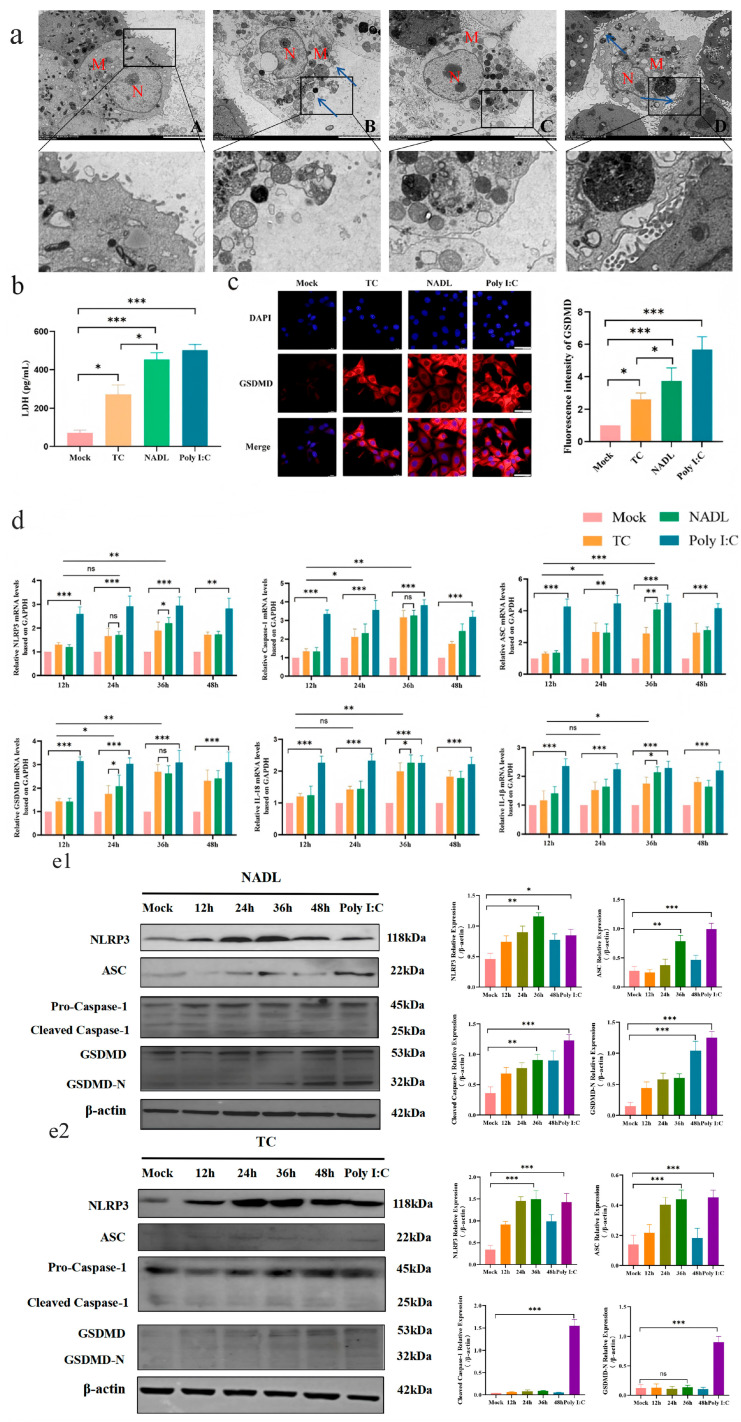
BVDV NADL induces NLRP3 inflammasome activation not TC strain; (**a**) Cells were infected at an MOI of 1 with the cytopathogenic BVDV strain NADL and the non-cytopathogenic TC strain. (**A**). Mock group; (**B**). BVDV NADL group; (**C**). BVDV TC group; (**D**). Poly I:C group M: mitochondria; N: nucleus; blue arrow: site of membrane rupture;Please confirm if it is needed to add the explanation for arrows and boxe; scale bar: 5.0 μm. (**b**) Cell viability was assessed by LDH assay after 36 h; (**c**) fluorescent expression of pyroptosis effector GSDMD detected by IFA. Scale bar: 50 μm. (**d**) mRNA expression levels of pyroptosis-related genes were detected using qPCR. (**e**) Expression levels of pyroptosis-related proteins were detected using Western blot. Data are expressed as mean ± SD. * *p* < 0.05; ** *p* < 0.01; *** *p* < 0.001. (See Supplementary Materials).

To further confirm the occurrence of pyroptosis, the ultrastructure of MDBK cells was examined by transmission electron microscopy. The results showed that infection with the cytopathogenic NADL strain disrupted cell membrane integrity, resulting in membrane rupture and cleft formation, as well as chromatin condensation within the nuclei. These morphological features were consistent with pyroptosis, whereas no significant changes were observed in cells infected with the non-cytopathogenic TC strain (Figure 2a). Subsequently, cellular damage was assessed using a lactate dehydrogenase (LDH) release assay, which revealed significantly elevated LDH levels in NADL strain-infected cells (Figure 2b). Immunofluorescence analysis of the pyroptosis effector protein GSDMD expression showed red fluorescence in the cytoplasm of NADL strain-infected cells, whereas no significant red fluorescence was observed in TC strain-infected cells (Figure 2c).

qPCR analysis of mRNA expression levels of pyroptosis-related genes revealed significant upregulation of the NLRP3 inflammasome complex (NLRP3, ASC, and Caspase-1), the pyroptosis executor protein (GSDMD), and downstream inflammatory cytokines (IL-1β and IL-18) (Figure 2d). Given that qPCR only reflects gene transcription levels and cannot assess the processing and functional activation status of key pyroptosis proteins, Western blot analysis was further employed in this study to examine the expression and cleavage patterns of key proteins in the NLRP3 inflammasome pathway. Results demonstrated that NADL strain infection induced a time-dependent upregulation of NLRP3 and ASC protein levels in cells. Simultaneously, NADL strain infection significantly induced the functional cleavage of Caspase-1 and GSDMD, yielding Caspase-1 p20 and GSDMD-N fragments, respectively (Figure 2(e1,e2)). In contrast, no significant cleaved fragments of Caspase-1 or GSDMD were detected in cells infected with the TC strain (Figure 2(e1,e2)). These findings indicate that the BVDV NADL strain can effectively activate the NLRP3 inflammasome downstream effector pathway, thereby inducing Caspase-1 and GSDMD-mediated pyroptosis.

### 3.3. BVDV Core Protein C Induces Pyroptosis in MDBK Cells

**Figure 3:**
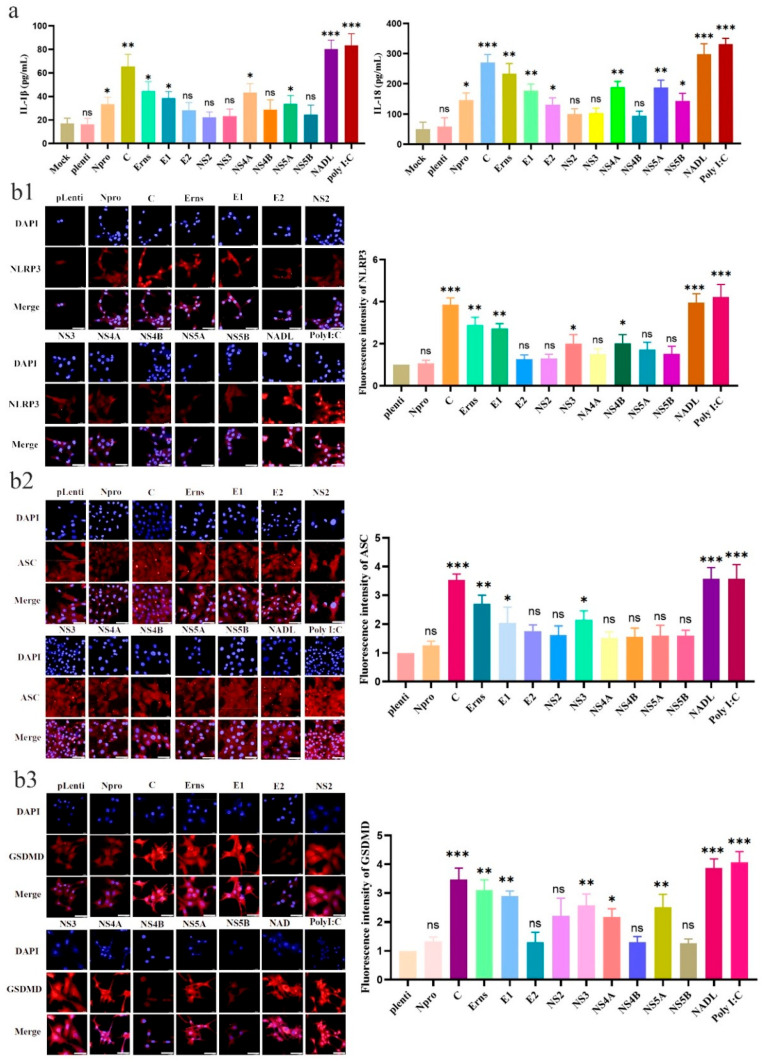
BVDV core protein C induces pyroptosis in MDBK cells; (**a**) infection of MDBK cells with a lentivirus encoding the NADL strain protein at an MOI of 5 for 36 h, followed by detection of pro-inflammatory factor secretion; (**b**) fluorescent expression of NLRP3, ASC, and GSDMD detected by IFA 36 h after lentiviral infection, (**b1**) NLRP3 protein, (**b2**) ASC protein, and (**b3**) GSDMD protein were detected, respectively. Scale bar: 50 μm. Data are presented as mean ± SD. * *p* < 0.05; ** *p* < 0.01; *** *p* < 0.001.

Infection of MDBK cells with the BVDV NADL strain can further induce pyroptosis by activating the NLRP3 inflammasome. Previous studies generally suggest that virus-induced pyroptosis typically relies on viral-encoded proteins to mediate host inflammasome activation and programmed cell death processes. Therefore, this section of the study employed ELISA to detect IL-1β and IL-18 secretion in the supernatants following infection of MDBK cells with recombinant lentiviruses encoding 11 BVDV NADL strain proteins (Npro, C, Erns, E1, E2, NS2, NS3, NS4A, NS4B, NS5A, and NS5B). Results indicate that IL-1β and IL-18 secretion levels were highest in cells expressing the C protein (Figure 3a). Immunofluorescence assay (IFA) analysis revealed significant expression levels of the inflammasome component proteins NLRP3, ASC, and GSDMD in the C protein group as well (Figure 3(b1–b3)). Although several structural proteins induced varying degrees of inflammasome activation, quantitative analysis demonstrated that the C protein elicited the strongest response. Therefore, the C protein was selected for subsequent mechanistic studies.

### 3.4. BVDV Core Protein Binds with ASC to Induce Inflammasome Assembly and Pyroptosis

**Figure 4:**
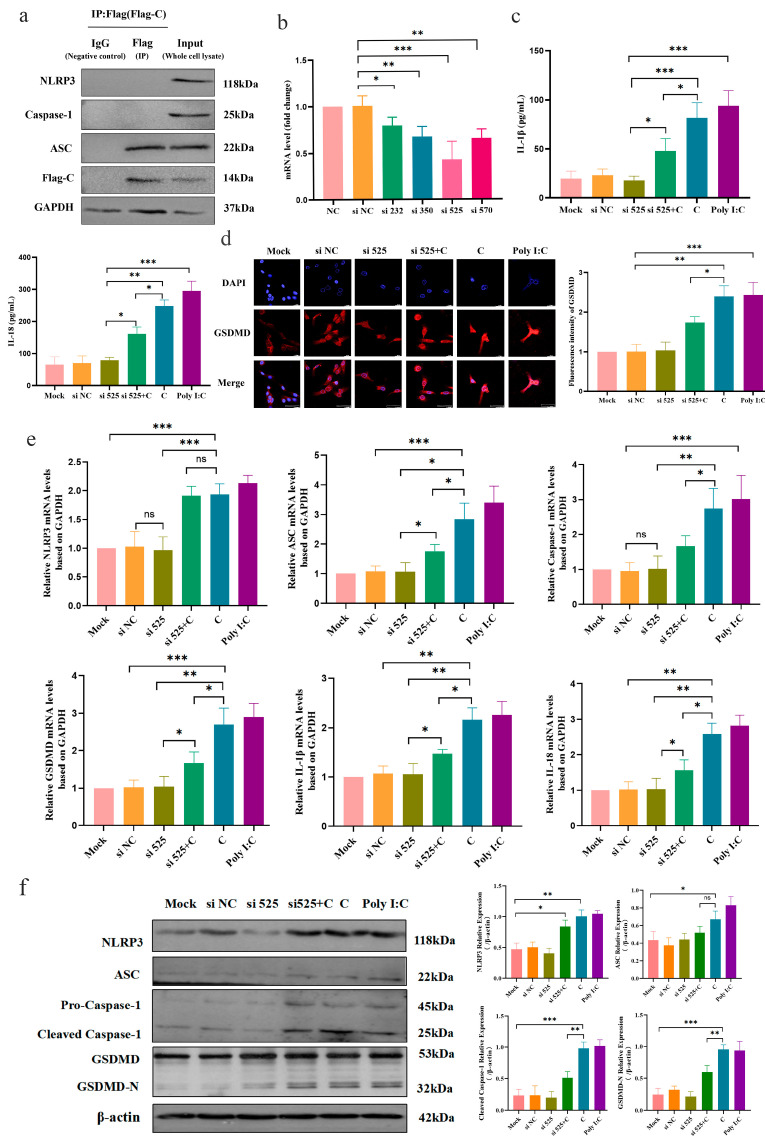
BVDV core protein binds with ASC to induce inflammasome assemble and pyroptosis; (**a**) Flag-tagged C protein was overexpressed in MDBK cells, where NLRP3, ASC, and Caspase-1 were labeled with specific antibodies. Co-immunoprecipitation (Co-IP) analysis was performed to investigate the interaction between viral protein C and components of the inflammasome complex. Input represents the whole-cell lysates before immunoprecipitation. IP indicates proteins immunoprecipitated with anti-Flag antibody. IgG was used as a negative control. (**b**) The interference efficiency of the four synthesized siRNAs (si-232, si-350, si-525, and si-570) was screened by qPCR; (**c**) ELISA assay was use to detect pro-inflammatory cytokine secretion in the cell supernatants following ASC knockdown; (**d**) IFA was used to detect GSDMD protein expression in cells after ASC knockdown, scale bar: 50 μm; (**e**) detection of mRNA expression levels of pyroptosis-related genes following ASC knockdown using qPCR; (**f**) detection of protein expression levels of pyroptosis-related genes following ASC knockdown using Western blot analysis. Statistical analysis of NLRP3, caspase-1, and GSDMD protein expression between the si525+C and C groups was performed using one-way ANOVA followed by Tukey’s multiple comparisons test. Data are presented as mean ± SD. * *p* < 0.05; ** *p* < 0.01; *** *p* < 0.001. (See Supplementary Materials).

Given that the NLRP3 inflammasome is composed of three key components, NLRP3, ASC, and Caspase-1, its complete assembly and functional activation depend on precise interactions among these components. Therefore, systematically elucidating the interactions between the core protein C of the BVDV NADL strain and the various components of the NLRP3 inflammasome is crucial for gaining a deeper understanding of the molecular mechanisms mediating pyroptosis. Co-immunoprecipitation (Co-IP) was used to analyze the relationship between viral protein C and inflammasome complex components, which revealed that protein C interacts exclusively with ASC in MDBK cells, but not with NLRP3 or Caspase-1 (Figure 4a). This indicates that BVDV core protein C interacts with ASC of the NLRP3 inflammasome. To further validate the interaction between BVDV core protein C and ASC, which subsequently activates the NLRP3 inflammasome, this study targeted ASC with synthetic siRNA. qPCR results indicated that si-525 reduced ASC mRNA expression by approximately 50% (Figure 4b), which was consistent with a partial reduction in ASC protein expression (Figure 4f). Using an ELISA kit, we assessed pro-inflammatory cytokine secretion in cell supernatants after BVDV core protein C lentiviral infection in ASC-interfered cells. Results demonstrated that ASC gene interference significantly reduced IL-1β and IL-18 secretion (Figure 4c). Concurrently, immunofluorescence analysis revealed that ASC interference effectively suppressed the expression of pyroptosis effector protein GSDMD (Figure 4d).

To further investigate the effects of BVDV core protein C on pyroptosis in MDBK cells following ASC knockdown, qPCR was employed to detect mRNA expression levels of pyroptosis-related genes after ASC knockdown and subsequent infection with a lentivirus-expressing BVDV core protein C. Results demonstrated that interfering with ASC protein expression significantly reduced mRNA expression levels of Caspase-1, ASC, GSDMD, IL-1β, and IL-18 mRNA expression, while NLRP3 mRNA levels remained unaffected (Figure 4e). Western blot analysis showed that si525+C treatment did not significantly affect NLRP3 expression but reduced caspase-1 and GSDMD levels compared with the C group, suggesting that ASC knockdown mainly affects downstream inflammasome activation and pyroptosis-related proteins (Figure 4f). These findings indicate that interfering with ASC protein expression effectively suppressed BVDV core protein C-induced secretion of IL-1β and IL-18 in MDBK cells, as well as the cleavage and activation of pro-Caspase-1 and GSDMD, thereby inhibiting pyroptosis.

## 4. Discussion

Bovine viral diarrhea virus (BVDV) is an economically important pathogen that substantially affects the global cattle industry, and can significantly reduce animal productivity, resulting in an economic loss of approximately $61.57 per cow per year (converted from £46.50 according to the exchange rate) [27,28]. Given its widespread prevalence and the severe impact it causes, it has been classified by the World Organisation for Animal Health (WOAH) as a Category B infectious animal disease [29]. Currently, BVDV continues to be prevalent in 88 countries and regions worldwide, underscoring its efficient transmission capability and the substantial epidemiological risk [26]. Therefore, systematically analyzing the molecular characteristics and regulatory networks of BVDV-induced pyroptosis in MDBK cells is of crucial scientific value for elucidating the pathogenic mechanisms of the virus and revealing the regulatory mechanisms of the host inflammatory response.

The concept of cell pyroptosis was first proposed by the American scholar Brad T. Cookson in 2001 based on observations of macrophages infected by pathogens, revealing the molecular basis by which innate immune cells achieve an active defense through programmed inflammatory death [30]. Recent studies have further expanded this understanding, confirming that various non-immune cells, including epithelial cells [31,32], endothelial cells [33,34] and organ parenchymal cells [35,36], also possess the complete molecular mechanism for pyroptosis execution. This has established the key regulatory role of this mode of death in the occurrence and progression of infectious diseases. However, in the process of bovine viral diarrhea virus (BVDV) infecting bovine kidney epithelial cells (MDBK), whether pyroptosis is activated and its regulatory mechanisms still lack systematic research. To our knowledge, this study is the first to demonstrate that BVDV can induce pyroptosis in MDBK cells; the ELISA and IFA results showed that both the NADL strain and the TC strain can upregulate the expression of inflammatory factors such as IL-1β and IL-18, and promote the co-localization of NLRP3, ASC, and Caspase-1. This finding is in line with the results reported by Pandey et al. in their study on pathogen-induced inflammasome assembly. It indicates that both biotypes of BVDV have the ability to activate the upstream signals of the NLRP3 inflammasome [37]. However, further ultrastructural and functional analyses revealed the fundamental differences between the two strains in inducing pyroptosis: only the NADL strain can induce the formation of typical pyroptotic pores in the cell membrane and significantly exacerbate cell damage, whereas the TC strain, although able to activate some inflammatory signals, fails to cause obvious membrane rupture. This phenomenon is highly consistent with the research findings of Li and Weir [38,39]. These results indicate that inflammasome signaling activation and the transcriptional upregulation of inflammatory mediators alone are insufficient to trigger pyroptosis. The actual execution of pyroptosis must rely on the effective cleavage of the GSDMD protein and the transmembrane pore formation it mediates. Only when the integrity of the cell membrane is compromised can the pyroptosis program be fully activated [40,41]. It is worth noting that although both strains can upregulate the expression of NLRP3 inflammasome-related genes at the transcriptional level, only the CP-type NADL strain can effectively induce GSDMD cleavage and initiate downstream pyroptosis programs. This difference indicates that CP-type BVDV tends to fully activate the inflammasome effector axis, triggering an acute inflammatory response through the induction of lytic cell death, whereas NCP-type BVDV is blocked at the execution phase of pyroptosis. This phenomenon may help the virus avoid the rapid lysis of host cells, thereby creating favorable conditions for the establishment of persistent infection (PI) [42,43].

Previous studies have shown that various viral structural proteins, in addition to completing virus assembly and replication, can also act as immune regulatory factors directly involved in the regulation of host inflammasome signaling pathways [44]. For example, the influenza virus M2 protein activates the NLRP3 inflammasome by disrupting intracellular ion homeostasis [45], and the hepatitis B virus core protein can trigger downstream inflammatory responses [46]. The Zika virus non-structural protein NS5 has been shown to target inflammasome components to amplify inflammatory signals [47]. These findings collectively reveal that the precise regulation of inflammasome pathways by virus-encoded proteins is often the key factor determining whether an infection progresses toward acute inflammation or immune evasion. However, as an important pathogen of the cattle family, the direct role of BVDV-encoded proteins in inflammasome activation and pyroptosis regulation has previously lacked systematic evidence. This study found that the core protein C of the BVDV NADL strain can significantly promote the secretion of IL-1β and IL-18 and upregulate the expression of NLRP3, ASC, and GSDMD, confirming its ability to activate the inflammasome and drive pyroptosis, thereby providing new evidence for understanding the molecular mechanism of acute inflammation induced by CP BVDV. Further mechanistic analysis showed that the core protein C does not induce an inflammatory response through nonspecific cellular damage, but rather promotes the effective assembly of the NLRP3 inflammasome through specific interaction with ASC, thereby activating downstream pyroptotic processes in an ASC-dependent manner. Specifically, after interfering with ASC expression, both the secretion of inflammatory factors induced by the core protein C and the upregulation of molecules related to pyroptosis were significantly weakened, confirming the pivotal role of ASC in this process. It is worth noting that the regulation mode of the core protein C targeting ASC is not an isolated case. Zhang et al. [48] reported that the Pseudorabies virus UL4 protein also activates NLRP3 and AIM2 inflammasomes by targeting ASC, suggesting that ASC may be an inflammasome regulatory node commonly targeted by multiple viral proteins. As a key adaptor protein, ASC plays a central role in inflammasome assembly and signal amplification, and has both PYD and CARD domains, allowing it to bridge upstream sensors and downstream effector protein Caspase-1 [49,50]. Viral proteins can effectively modulate inflammatory signaling pathways by targeting ASC, which can either amplify inflammation to promote viral spread or suppress inflammation to establish persistent infection.

Although MDBK cells provide a useful in vitro model for investigating BVDV-induced inflammatory responses, this study has several limitations. MDBK cells are derived from bovine kidney epithelial cells and cannot fully represent the complex immune environment and tissue interactions occurring during natural BVDV infection. The extent to which NADL-induced ASC-dependent NLRP3 inflammasome activation and GSDMD-mediated pyroptosis occur in naturally infected cattle remains to be further investigated. As kidney epithelial cells can serve as important target cells during BVDV infection and may contribute to virus replication and host inflammatory responses, our findings suggest that renal epithelial cell-mediated inflammasome activation may participate in BVDV-associated tissue injury and disease progression. Future studies using primary bovine kidney epithelial cells, organoid models, and animal infection experiments are needed to validate whether this mechanism contributes to BVDV pathogenesis in vivo. In addition, analyzing inflammasome activation and pyroptotic markers in tissues from BVDV-infected cattle will provide further evidence for the physiological relevance of this pathway.

This study reveals the key molecular differences in the pathogenic mechanisms between cytopathogenic and non-cytopathogenic BVDV from the perspective of inflammasome-mediated pyroptosis, However, certain limitations persist. Firstly, this study primarily utilized an in vitro MDBK cell model, which may not fully recapitulate the dynamic regulation of inflammasome signals in the complex immune microenvironment in vivo. Secondly, beyond the NLRP3 inflammasome, the potential roles of other types of inflammasomes and their cross-regulatory effects in BVDV infection warrant further investigation. Additionally, the specific structural basis of the interaction between BVDV core protein C and ASC and its precise regulatory mechanism likewise require verification through more in-depth structural biology studies and in vivo functional experiments. Moving forward, research could leverage animal infection models and gene editing technology to systematically evaluate the dual roles of the inflammasome–pyroptosis pathway in BVDV pathogenesis and host antiviral immunity, as well as explore intervention strategies targeting this pathway. In conclusion, this study not only deepens our understanding of the regulatory mechanisms of inflammatory responses during BVDV infection, but also provides important theoretical insights for the development of new antiviral control measures.

## 5. Conclusions

**Figure 5:**
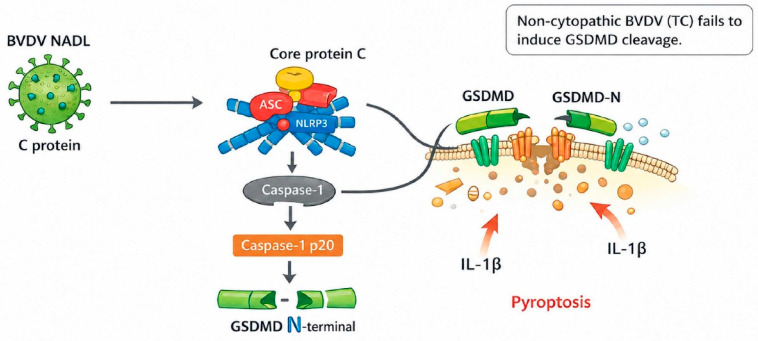
Simplified model of BVDV-induced pyroptosis via ASC-dependent NLRP3 inflammasome activation.

This study investigates mechanisms of pyroptosis mediated by inflammasomes and systematically analyzes the pathogenic differences in different BVDV biotypes. Our findings demonstrate that the cytopathogenic variant NADL strain specifically interacts with the adaptor protein ASC via its core protein C, leading to NLRP3 inflammasome assembly and Caspase-1 activation. This subsequently mediates GSDMD cleavage, membrane pore formation, and ultimately, pyroptosis. Non-cytopathogenic TC strains can activate upstream signals of the inflammasome, but are insufficient to drive the complete pyroptosis pathway. These findings collectively suggest that the BVDV core protein C-ASC axis serves as a key molecular switch regulating inflammasome activation and pyroptosis, thereby dictating the distinct outcomes of infection with different biotypes of the virus. This study elucidates the key molecular of BVDV-induced host inflammatory damage at the molecular level and provides a theoretical basis for intervention strategies targeting the inflammasome pathway (Figure 5).

## Data Availability

The original contributions presented in this study are included in the article/Supplementary Materials. Further inquiries can be directed to the corresponding authors.

## Supplementary Materials

The following supporting information can be downloaded at: https://www.mdpi.com/article/10.3390/vetsci13070673/s1, Uncropped Western blot images corresponding to the Western blot experiments presented in the main text.
