# Supplementary figures and images for: The Cytopathogenic BVDV Core Protein Binds with ASC-Enhance the Assembly of Inflammasome Complex and GSDMD-Mediated Pyroptosis
Source: Vet Sci. 2026 Jul 10;13(7):673. doi: 10.3390/vetsci13070673 (PMC13431418; doi:10.3390/vetsci13070673)

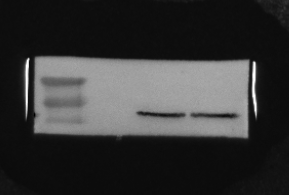

Supplement: Supplementary file 1 [file vetsci-13-00673-s001.zip › Figure 4/a/ASC.tif]

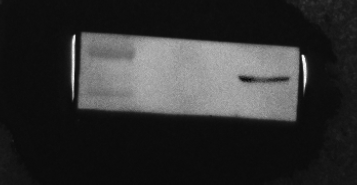

Supplement: Supplementary file 1 [file vetsci-13-00673-s001.zip › Figure 4/a/Caspase-1.tif]

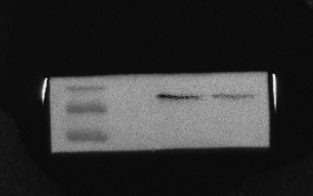

Supplement: Supplementary file 1 [file vetsci-13-00673-s001.zip › Figure 4/a/Flag-C.tif]

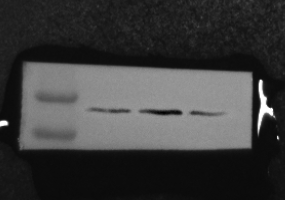

Supplement: Supplementary file 1 [file vetsci-13-00673-s001.zip › Figure 4/a/GAPDH.tif]

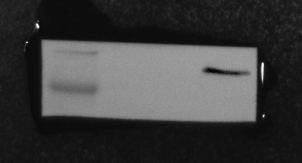

Supplement: Supplementary file 1 [file vetsci-13-00673-s001.zip › Figure 4/a/NLRP3.tif]

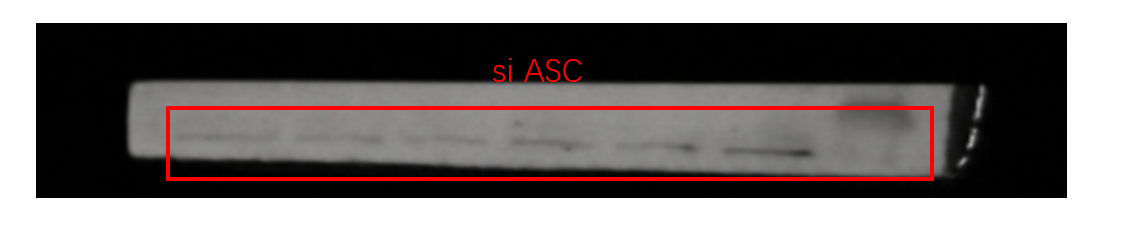

Supplement: Supplementary file 1 [file vetsci-13-00673-s001.zip › Figure 4/f/ASC.png]

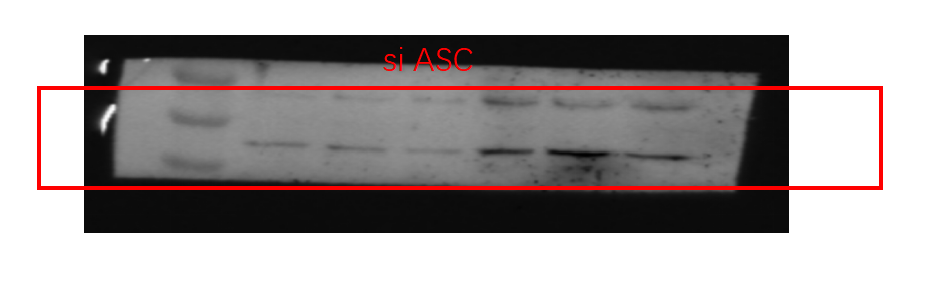

Supplement: Supplementary file 1 [file vetsci-13-00673-s001.zip › Figure 4/f/Caspase-1.png]

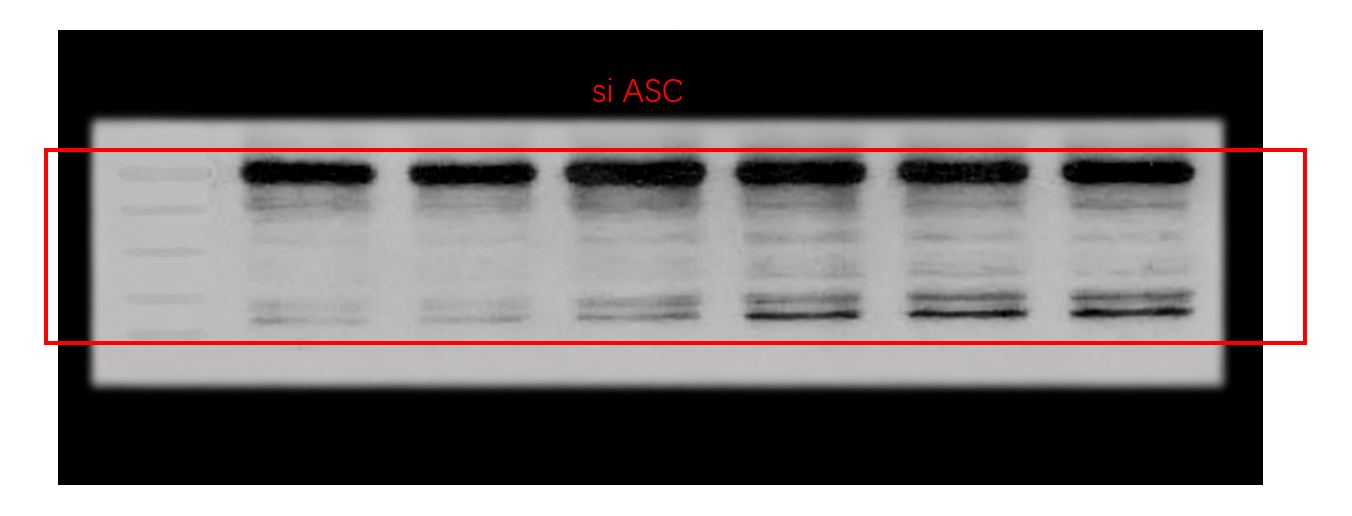

Supplement: Supplementary file 1 [file vetsci-13-00673-s001.zip › Figure 4/f/GSDMD.png]

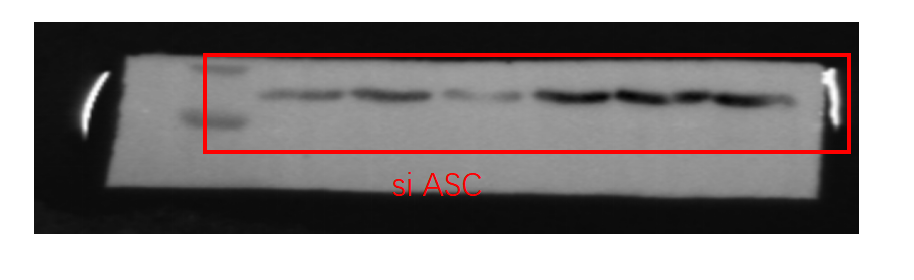

Supplement: Supplementary file 1 [file vetsci-13-00673-s001.zip › Figure 4/f/NLRP3.png]

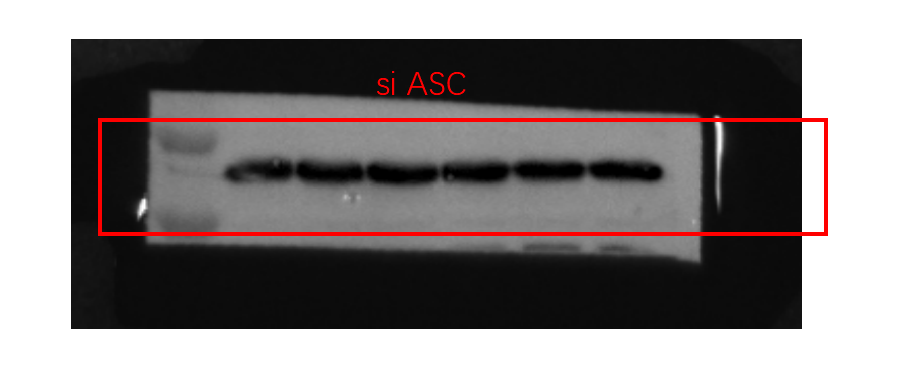

Supplement: Supplementary file 1 [file vetsci-13-00673-s001.zip › Figure 4/f/β-actin.tif]

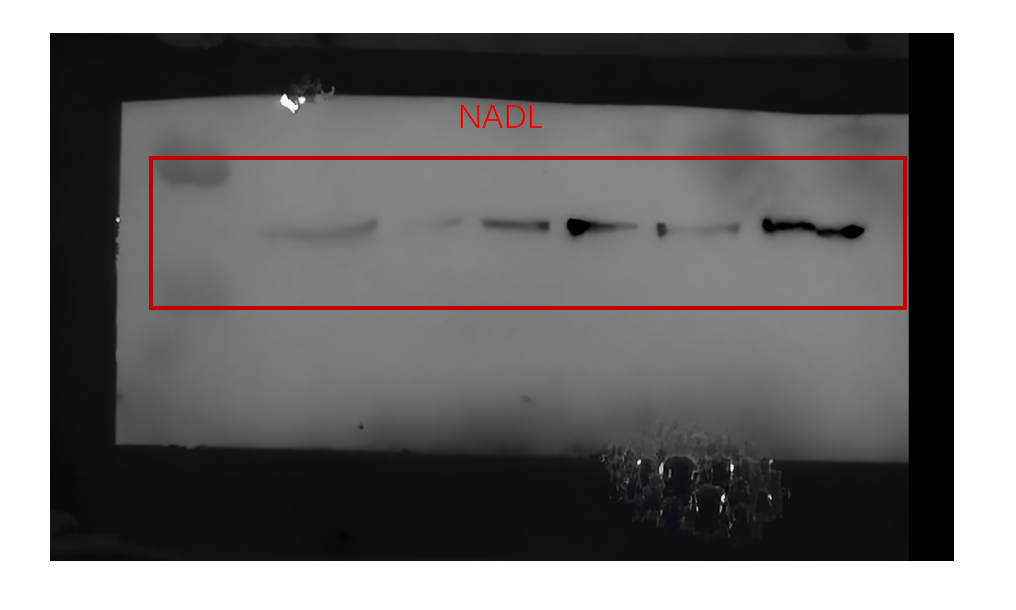

Supplement: Supplementary file 1 [file vetsci-13-00673-s001.zip › Figure 2/e1/ASC.png]

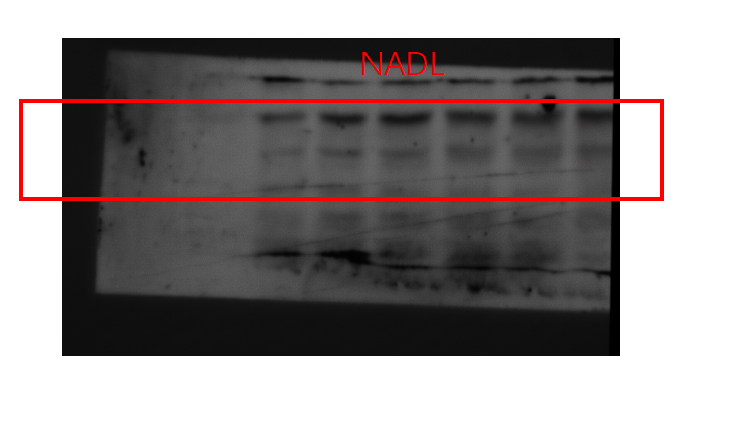

Supplement: Supplementary file 1 [file vetsci-13-00673-s001.zip › Figure 2/e1/Caspase-1.png]

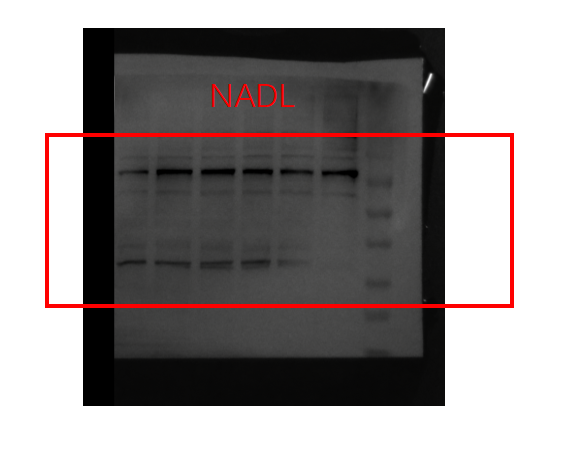

Supplement: Supplementary file 1 [file vetsci-13-00673-s001.zip › Figure 2/e1/GSDMD.png]

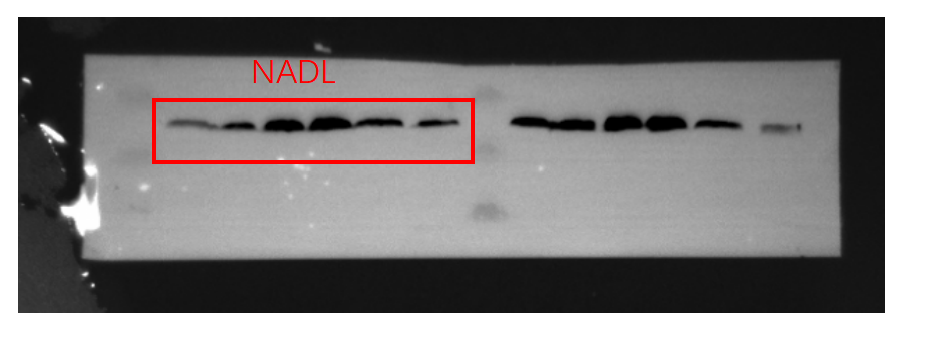

Supplement: Supplementary file 1 [file vetsci-13-00673-s001.zip › Figure 2/e1/NLRP3(NADL).png]

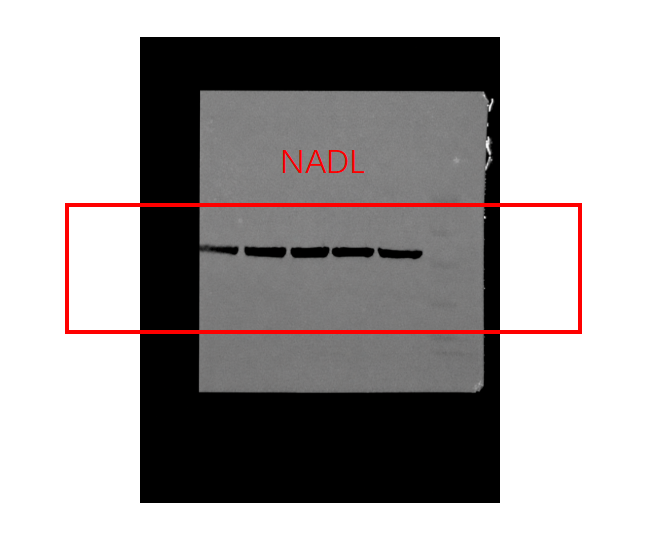

Supplement: Supplementary file 1 [file vetsci-13-00673-s001.zip › Figure 2/e1/β-actin.png]

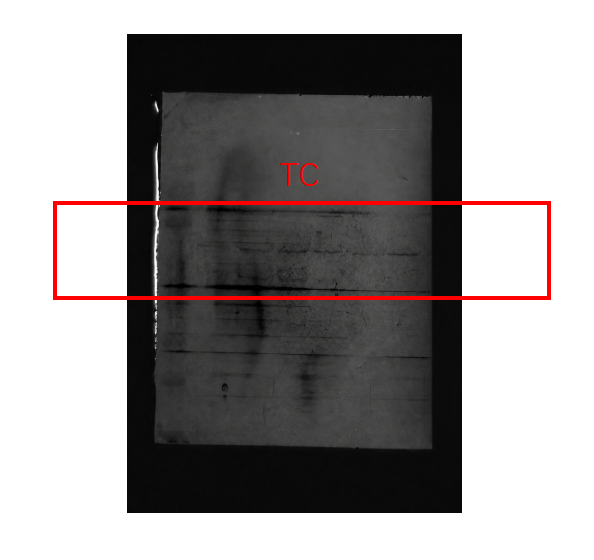

Supplement: Supplementary file 1 [file vetsci-13-00673-s001.zip › Figure 2/e2/ASC.png]

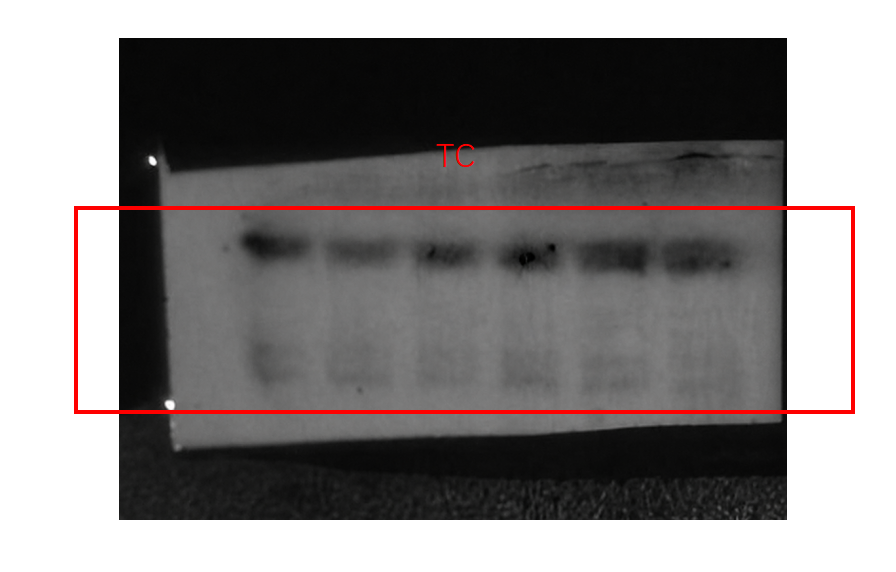

Supplement: Supplementary file 1 [file vetsci-13-00673-s001.zip › Figure 2/e2/Caspase-1.png]

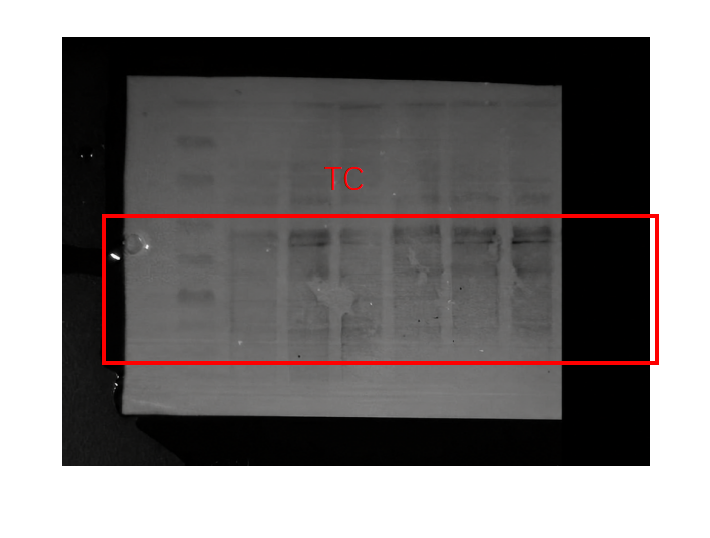

Supplement: Supplementary file 1 [file vetsci-13-00673-s001.zip › Figure 2/e2/GSDMD.png]

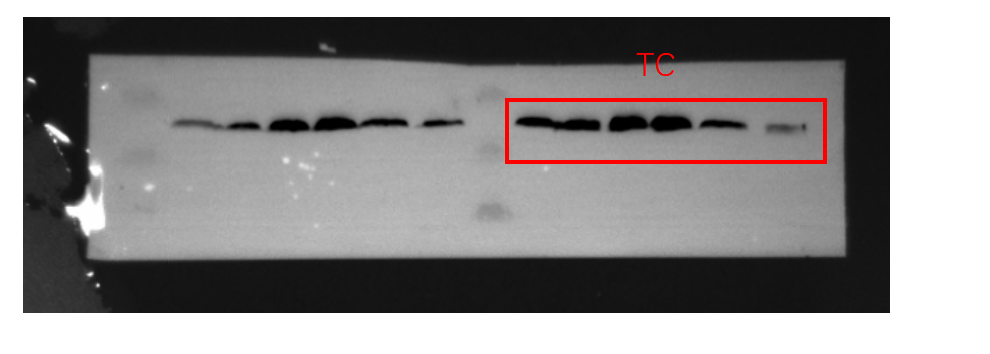

Supplement: Supplementary file 1 [file vetsci-13-00673-s001.zip › Figure 2/e2/NLRP3(TC).png]

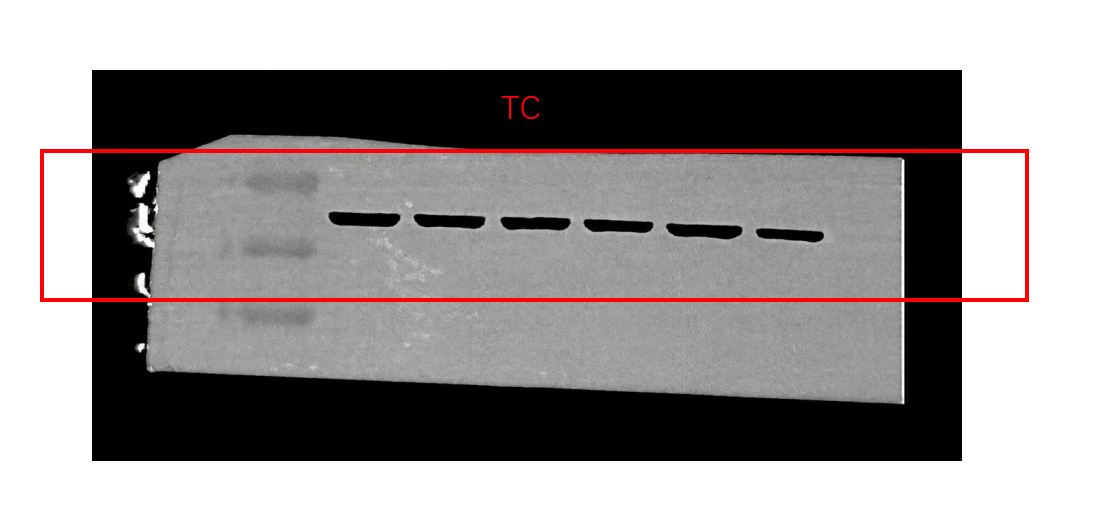

Supplement: Supplementary file 1 [file vetsci-13-00673-s001.zip › Figure 2/e2/β-actin.png]
